# β1 Integrin Maintains Integrity of the Embryonic Neocortical Stem Cell Niche

**DOI:** 10.1371/journal.pbio.1000176

**Published:** 2009-08-18

**Authors:** Karine Loulier, Justin D. Lathia, Veronique Marthiens, Jenne Relucio, Mohamed R. Mughal, Sung-Chun Tang, Turhan Coksaygan, Peter E. Hall, Srinivasulu Chigurupati, Bruce Patton, Holly Colognato, Mahendra S. Rao, Mark P. Mattson, Tarik F. Haydar, Charles ffrench-Constant

**Affiliations:** 1 Center for Neuroscience, Children's National Medical Center, Washington, D.C., United States of America; 2 Department of Pathology, University of Cambridge, Cambridge, United Kingdom; 3 Department of Medical Genetics, University of Cambridge, Cambridge, United Kingdom; 4 Centre for Brain Repair, University of Cambridge, Cambridge, United Kingdom; 5 Laboratory of Neuroscience, National Institute on Aging Intramural Research Program, Baltimore, Maryland, United States of America; 6 Department of Pharmacology, State University of New York at Stony Brook, Stony Brook, New York, United States of America; 7 School of Medicine, University of Maryland, Baltimore, Maryland, United States of America; 8 Center for Research on Occupational and Environmental Toxicology, Oregon Health and Science University, Portland, Oregon, United States of America; 9 Corporate Research Laboratories, Invitrogen Corporation, Carlsbad, California, United States of America; 10 Department of Neuroscience, Johns Hopkins University School of Medicine, Baltimore, Maryland, United States of America; Univ. North Carolina, United States of America

## Abstract

During embryogenesis, the neural stem cells (NSC) of the developing cerebral cortex are located in the ventricular zone (VZ) lining the cerebral ventricles. They exhibit apical and basal processes that contact the ventricular surface and the pial basement membrane, respectively. This unique architecture is important for VZ physical integrity and fate determination of NSC daughter cells. In addition, the shorter apical process is critical for interkinetic nuclear migration (INM), which enables VZ cell mitoses at the ventricular surface. Despite their importance, the mechanisms required for NSC adhesion to the ventricle are poorly understood. We have shown previously that one class of candidate adhesion molecules, laminins, are present in the ventricular region and that their integrin receptors are expressed by NSC. However, prior studies only demonstrate a role for their interaction in the attachment of the basal process to the overlying pial basement membrane. Here we use antibody-blocking and genetic experiments to reveal an additional and novel requirement for laminin/integrin interactions in apical process adhesion and NSC regulation. Transient abrogation of integrin binding and signalling using blocking antibodies to specifically target the ventricular region in utero results in abnormal INM and alterations in the orientation of NSC divisions. We found that these defects were also observed in laminin α2 deficient mice. More detailed analyses using a multidisciplinary approach to analyse stem cell behaviour by expression of fluorescent transgenes and multiphoton time-lapse imaging revealed that the transient embryonic disruption of laminin/integrin signalling at the VZ surface resulted in apical process detachment from the ventricular surface, dystrophic radial glia fibers, and substantial layering defects in the postnatal neocortex. Collectively, these data reveal novel roles for the laminin/integrin interaction in anchoring embryonic NSCs to the ventricular surface and maintaining the physical integrity of the neocortical niche, with even transient perturbations resulting in long-lasting cortical defects.

## Abstract 2

IInteractions between laminins and integrin receptors hold neural stem cells in place at the ventricular surface of embryonic brain. Transient disruption leads to abnormal stem cell divisions and permanent cortical malformation.

## Author Summary

The developing cerebral cortex contains bipolar neural stem cells that span the cortical layers between the inner ventricular surface and the outer pial surface of the embryonic brain. The nuclei of these cells remain near the ventricular cavity, a microenvironment or niche thought to provide vital signals. It is not known how this inner end of the bipolar stem cell is held in place, or what would happen if its attachment to the inner surface were lost. Genetic manipulation can be used to disrupt candidate molecules involved in this adhesion, but this will affect all adhesion points and complicate the results. We have therefore developed an approach to target the stem cell attachments specifically at the ventricular surface by placing blocking antibodies directly into the ventricles of mouse embryos and then expressing fluorescent markers in the stem cells to see the effects of losing this attachment in living tissue. We examined laminins and integrins, whose expression and properties make them excellent candidates. Blocking integrin signalling by antibody application caused the inner end of the stem cells to rapidly detach and then undergo aberrant cell division. We also showed that a transient block of integrins (for <2 hours) resulted in permanent malformations of the cortical layers and disrupted neuronal migration.

## Introduction

The cues responsible for maintaining the physical and molecular architecture of the stem cell niche of the developing mammalian brain are not well known. In the mammalian neocortex, the radial glia neural stem cells (NSC) that generate neurons are bipolar and have a radial morphology that spans the developing neocortical wall [1],[2]. These NSC have their soma located within the ventricular zone (VZ) adjacent to the ventricle, and their apical and basal processes make contact with the ventricular surface and the pial basement membrane, respectively [3],[4]. The basal (pial) process is important for informing the fate of the NSC daughter cells and then acting as a guidepost for their migration [5]–[7]. In contrast, the apical process contains cilia that extend into the ventricle and are thought to be important for morphogen signalling within the VZ microenvironment or niche [8],[9]. In addition, the apical process is necessary for the interkinetic nuclear migration (INM), which takes place during VZ cell proliferation [4],[10], and its transection results in translocation of the NSC soma away from the ventricular surface [7]. The apical processes of adjacent radial glia cells are attached to one another via cadherin-based adherens junctions that are Numb and Numbl-dependant [11],[12]. Recent studies have also highlighted the importance of Cdc42 [13], αE-catenin [14], β-catenin [15],[16], and the adenomatous polyposis coli protein (APC) [17] in the maintenance of this morphology. Deletion of Cdc42 as well as Numb/Numbl in NSCs disrupts apical adherens junctions resulting in defects in cell proliferation and disorganized cortical lamination [12],[13]. Likewise, αE-catenin and β-catenin, components of adherens junctions, regulate NSC cell cycle progression and thereby cerebral cortical size [14]–[16],[18]. Recently, APC has been shown to regulate the development and maintenance of the radial glial scaffold during corticogenesis [17]. However the specific adhesive molecules required for anchorage of these interconnected apical processes within the ventricular niche and their impact on neocortical development have not yet been determined.

The integrin α6β1 heterodimer is expressed at high levels in the apical regions of NSC [19]. Laminins, which serve as ligands for integrins in the extracellular matrix (ECM), are also present in the VZ niche [19], suggesting a possible role of laminins and integrins in providing these adhesive signals for NSC within the VZ. However, previous studies examining conditional deletion of β1 integrin in NSC [20] or α6^−/−^ mice [21] did not report abnormalities of NSC behaviour in the VZ. We reasoned that this might reflect compensation for the long-term loss of one integrin by other heterodimer combinations, as has been described for β subunit integrin mutants in *Drosophila* midgut development [22]. To test the potential role of laminin/integrin binding in VZ maintenance and proliferation, we circumvented this possible compensation by transiently disrupting β1 integrin/laminin binding specifically in the VZ using blocking antibodies injected into the ventricle of the embryonic mouse brain. We also developed a novel ex vivo multiphoton time lapse imaging method that enables the effect of targeting of the blocking antibody to the cortical niche to be seen in real time. Furthermore, we analyzed VZ cell morphology and proliferation in laminin α2 deficient embryos. Together, our data demonstrate a novel role for laminin/integrin binding in the regulation of NSC proliferation and adhesion within the embryonic VZ, as well as its requirement to maintain the architecture of the neocortical niche.

## Results

### Specific Inactivation of β1 Integrin Function at the Ventricular Surface

**Figure 1:**
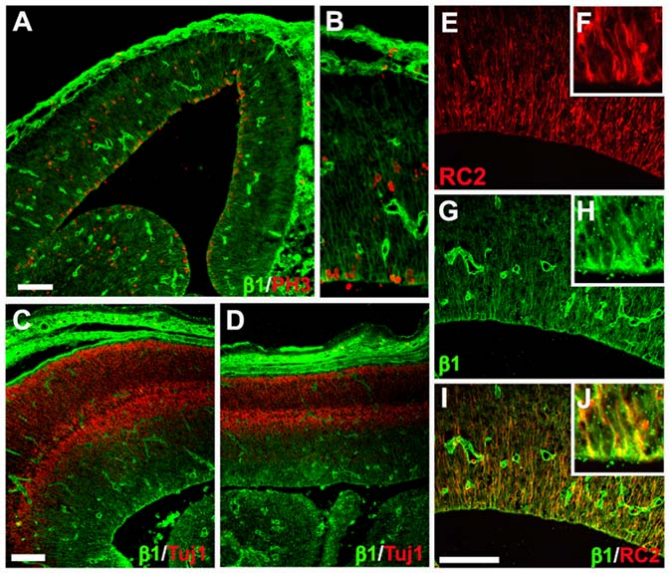
β1 integrin is expressed by radial glia and proliferating cells at the ventricular surface during neurogenesis. (A–J) Fluorescent micrographs of E13 coronal (A, B, E–J) or E16 sagittal (C, D) sections immunostained as indicated. Both at the rostral (A–C, E–J) and medial (D) levels, β1 integrin is expressed in PH3+ proliferating cells (A, high magnification B) and radial glia RC2+ cells (E–J) at the apical surface but not in Tuj1+ neurons (C, D). All scale bars represent 100 µm.

While β1 integrin (accession number Swiss Prot P09055, http://www.ebi.ac.uk/swissprot) has previously been shown to be present in the VZ of the developing cortex [19],[20],[23], we confirmed the expression levels in the neocortical wall on the embryonic days at which we performed the perturbation studies. At E13.5, there is a high level of β1 integrin in the VZ, as shown by double labelling with a mitotic marker of M-phase, phospho histone 3 (PH3, Figure 1A and 1B). The high level of β1 integrin continues into the cortical subventricular zone (SVZ) as marked by the second layer of PH3+ cells, and β1 integrin is also highly expressed at the pial surface and in blood vessels (Figure 1A and 1B). Importantly, there are particularly high levels of β1 integrin on the apical surface of the VZ and on radial glia apical fibers (as assessed by double labelling with RC2, Figure 1E–1J). Analysis of the subcellular localization of β1 integrin within the ventricular processes reveals that this receptor is mainly located immediately basal to the adherens junctions (Figure S1). At E16, as large numbers of neurons begin to differentiate in the cortex, the level of β1 integrin remains high in the VZ/SVZ but decreases in the neuronal layers (Figure 1C and 1D).

Because β1 integrin is also expressed at the pial surface, where it is involved in the organization of the cortical marginal zone [20], one major challenge was to preferentially inactivate β1 signalling only at the apical surface to determine the particular contribution of β1 integrin in the cellular dynamics that take place in the VZ during formation of the neocortical wall. To accomplish this, we delivered a blocking antibody (Ha2/5) [24] into the cerebral ventricle in utero to specifically block β1 integrin function in cells bordering the ventricle. To determine the efficacy of this approach, we first assessed the in vivo dynamics of the antibody by injecting fluorescently conjugated Ha2/5 into the ventricles of E14 mice in utero. We observed widespread localization of the antibody within the VZ and SVZ after 6 h (Figure S2B) and 24 h (unpublished data). The antibody penetration was confined to apical regions and did not reach the pial surface. To confirm in vivo that the antibody inhibited integrin signalling, we evaluated phospho-Akt 1 (p-Akt 1) levels in the cortex 30 min after injection. p-Akt-1 is known to be highly expressed in NSC and is a well-recognized downstream signalling molecule in the integrin pathway [25],[26]. Brain lysates were prepared from E12 and E15 embryos injected either with the Ha2/5 or with an isotype control (ITC) antibody. Western blotting revealed a reduced level of p-Akt 1 expression 30 min after injection (Figure S2C). By 2 h after antibody injection, the differences in p-Akt 1 levels were absent (unpublished data) demonstrating that the perturbation of β1 integrin signalling is transient.

### β1 Integrin Signalling Disruption at the Ventricular Surface Leads to Abnormalities of NSC Proliferation

**Figure 2:**
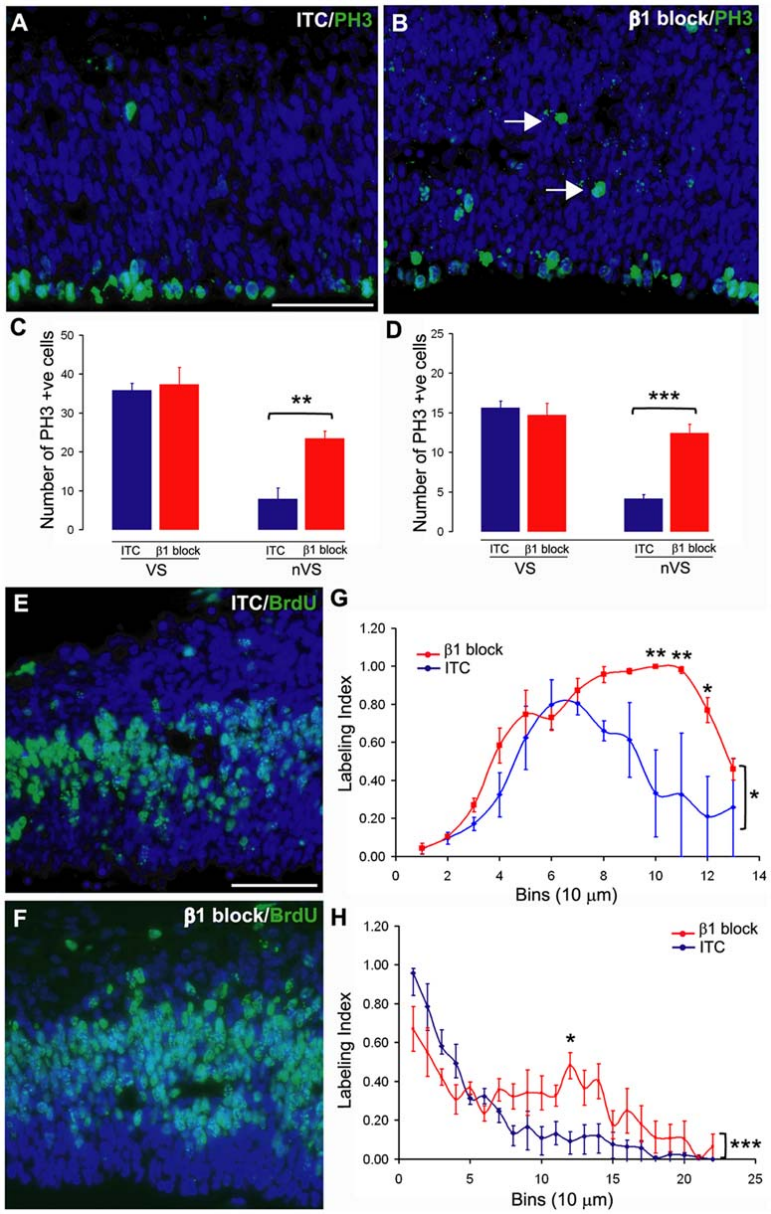
Cell proliferation is disrupted in the embryonic telencephalon after intraventricular injection of a β1 integrin blocking antibody. (A, B) Fluorescence micrographs of PH3 expression (green, dapi-counterstained nuclei in blue) after antibody injection at E12.5. Note the increase in PH3+ cells away from the ventricular surface in the β1 integrin blocking antibody-injected embryos (B), white arrows, compared to injected controls (A). (C, D) Quantification of PH3+ cells at the ventricular surface (VS) and nonventricular surface (nVS) after antibody injection at E12.5 (**, *p*<0.01 as assessed by a paired two-tailed *t*-test, C) and E15.5 (***, *p*<0.001, unpaired two-tailed *t*-test, D). (E, F) Fluorescence micrographs of BrdU expression (green, dapi-counterstained nuclei in blue) after antibody injection at E12.5, pulsed 1 h prior to sacrifice. Note the increase in the amount of BrdU+ cells in the telencephalon. (G, H) BrdU labelling index in E12.5- (G) and E15.5- (H) injected embryos pulsed with BrdU 1 h (G) or 6 h (H) prior to sacrifice. Note the increase in labelling index at 80–110 µm in the β1 integrin blocking antibody embryos, **, *p*<0.01 for bin 10 and 11, and *, *p*<0.05 for bin 12 calculated using Boneferroni post hoc tests. All scale bars represent 50 µm.

We took advantage of the movements of the NSC soma, which take place during cell cycle progression, to determine whether β1 integrin signalling affects the positioning of the NSC. Mitosis in the VZ normally occurs on the ventricular surface, after which the NSC soma transitions to the abventricular side of the VZ before entering S-phase (which can be identified by the incorporation of 5-bromo-2-deoxyuridine [BrdU] into the newly synthesized DNA). This cell cycle-dependent nuclear movement is known as INM [27]. Injection of 10 ng of the β1 integrin blocking antibody into the lateral ventricles of E12.5 and E15.5 embryos disrupted this pattern 18 h after injection, with mitotic PH3+ cells now scattered throughout the VZ (Figure 2A and 2B). Injection of a higher concentration of Ha2/5 (100 ng) produced identical results (unpublished data). Quantitative analysis (Figure 2C and 2D) revealed a significant increase in the number of PH3+ cells away from the ventricular surface (nonventricular surface or nVS) in both E12.5- (Figure 2C; *p*<0.01, unpaired two-tailed *t*-test) and E15.5-injected brains (Figure 2D; *p*<0.001, unpaired two-tailed *t*-test) without any changes seen in the number of PH3+ cells at the ventricular surface. Due to INM, a short 1 h pulse of BrdU normally labels cells clustered in S-phase at the abventricular boundary of the VZ (Figure 2E). Indeed, a maximum labelling index (calculated by the percentage of BrdU+ cells) was observed 60–70 µm away from the ventricular surface in the embryos injected with ITC antibodies (Figure 2G). In contrast, perturbation of β1 integrin signalling shifted the maximum labelling index 80–110 µm away from the ventricular surface (Figure 2F and 2G; *p*<0.05, two-way ANOVA), and also resulted in an overall increase in the number of BrdU+ cells. Thus in addition to the PH3+ mitotic cell ectopias, supernumerary S-phase cells were found in abnormal positions following β1 integrin blockade. To investigate somal translocation towards the VZ surface (i.e., M-phase reentry), E15.5-injected embryos were pulsed with BrdU 6 h prior to sacrifice, allowing the majority of proliferative cells to transit through S-phase and be on the ventricular surface either in or approaching mitosis at the time of analysis. In the embryos injected with the ITC antibody, this nuclear movement was indeed observed with the highest labelling index occurring in bin 1 nearest the ventricle (Figure 2H). While bin 1 also contained the highest labelling index in Ha2/5-injected brains, there was a significant increase in the labelling index of abventricular bins (bins 7–20) compared to controls. Thus, although continued ventricular divisions were apparent following blockade of β1 signalling, the abventricular dividing progenitor population was significantly increased.

**Figure 3:**
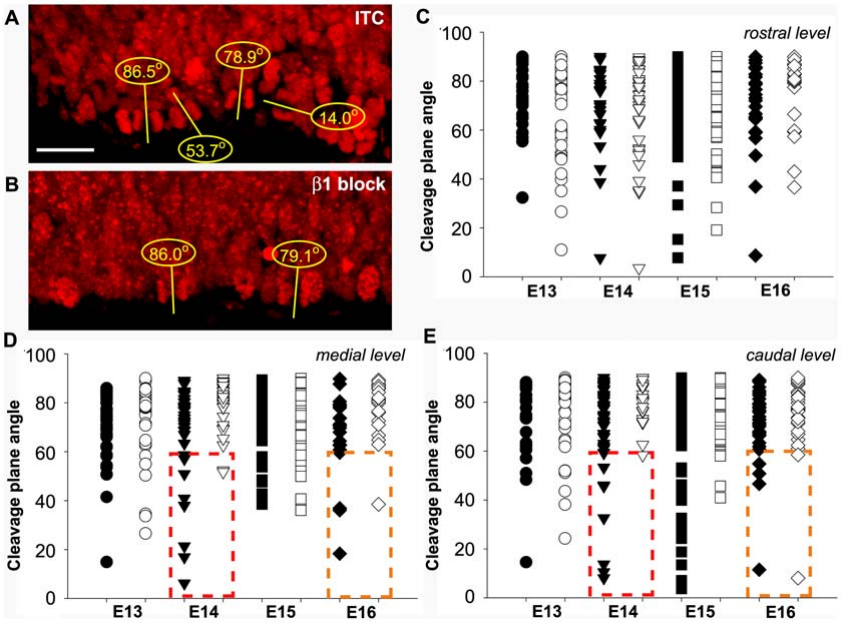
Intraventricular injection of β1 integrin blocking antibody prevents VZ horizontal mitotic cleavages throughout neurogenesis. (A, B) Micrographs of cells stained with propidium iodide in E14 telencephalon 18 h post ITC (A) or β1 integrin blocking antibody (B) injection. (C–E) Distribution of mitotic rostral (C), medial (D), and caudal (E) progenitors according to their angle of cleavage in ITC (black symbols) or β1 integrin blocking antibody (white symbols)-injected forebrains. Boxed regions highlight the paucity of horizontal cleavages (lower than 60 degrees) in Ha2/5-injected embryos. Scale bar represents 15 µm.

To determine whether changes in the SVZ may be related to the mode of division in the VZ, we analyzed the distribution of cleavage plane angles. It has previously been shown that the mitotic spindle undergoes significant rotation during metaphase [28]–[30], leading to changes in the cleavage orientation of mitotic figures, which may be an indication of cell fate [29]–[33]. In addition, β1 integrin signalling has previously been shown to affect mitotic spindle formation in Chinese Hamster Ovary (CHO) cells in vitro [34]. We therefore assessed the orientation of cell divisions in the VZ 18 h after antibody injection and found that the β1 integrin blocking antibody caused a significant change in the pattern of cell divisions (Figure 3). In the developing telencephalon, the majority of mitoses occur vertically with cleavage angles greater than 60 degrees relative to the ventricular surface [30],[31], although a small percentage (15%–20%) can be seen with lower degrees of cell division (i.e., horizontal divisions). Indeed, this is what was observed with the ITC-injected embryos 18 h after antibody injection on E12.5, E13.5, E14.5, and E15.5 at rostral, medial, and caudal levels of the telencephalon (Figure 3A, 3C–3E). However, there was a reduction in the amount of (horizontal) cell divisions with cleavage angles below 60 degrees in the β1 integrin antibody injected embryos in the medial and caudal regions of the dorsal telencephalon (Figure 3C–3E). Using a statistical model to analyze the distribution of the VZ cleavage angles throughout neurogenesis (from E13 to E16), we found that the proportion of horizontally dividing VZ cells (0–30 degrees) is significantly lower at the medial and caudal levels of the forebrain after disruption of β1 signalling (Figure S3A). Interestingly, the effect of blockade of β1 integrin signalling was not seen in neural precursors located rostrally (Figures 3C, 3E, and S3). Together, these data indicate that the ventricular divisions that remain following β1 integrin blockade exhibit altered cleavage parameters coincident with the increased number of abventricularly proliferating cells.

### Inactivation of β1 Integrin at the Ventricular Surface Does Not Lead to Premature Differentiation

Our BrdU and cell division studies clearly demonstrate that disruption of β1 integrin signalling leads to the presence of ectopic mitotic cells. We considered the possibility that these positioning defects lead to precocious differentiation. First, we determined the effects of β1 integrin blockade on the number of intermediate progenitor cells (IPC), which express the transcription factor T-brain 2 (Tbr2) [35]. IPC are neuronal progenitors that are generated from the NSC in the VZ, and which undergo further rounds of division just outside of the VZ in the SVZ [2],[35]. We found no difference in the number of Tbr2+ cells between ITC and Ha2/5-injected brains at E13 (Figure S4A–S4D). In addition, no premature neuronal (β3 tubulin, Figure S4E and S4F) or glial (NG2, unpublished data) differentiation was detected in the neocortical wall. Thus, β1 integrin blockade did not lead to abnormalities in cell differentiation within 18 h, and notably, although the number of proliferating cells in abventricular positions was increased, we found no increase in the number of Tbr2+ IPCs.

### Blockade of β1 Integrin Signalling at the Ventricular Surface Results in VZ Cell Detachment

**Figure 4:**
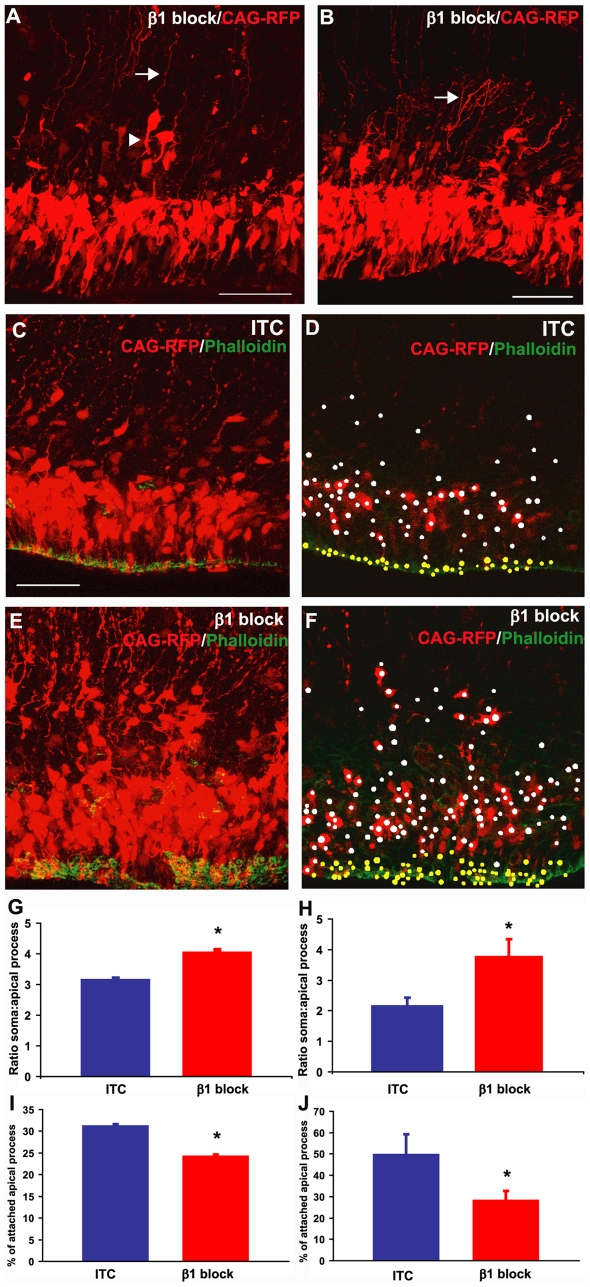
Inhibition of β1 integrin signalling results in NSC detachment. (A–F) Confocal fluorescence micrographs showing the VZ/SVZ of embryos injected at E15.5 with either an ITC (C, D) or β1 integrin blocking antibody (A, B, E, F) and simultaneously electroporated with CAG-RFP (red) and stained for phalloidin (green) 18 h later. (A) Note the bipolar morphology of the cell marked with an arrowhead and the dystrophic basal processes of detached cells (white arrows in A and B). (D, F) White and yellow dots represent soma and apical processes, respectively. (G, H) Quantification of the ratio of soma to apical processes in the co-injected/electroporated mouse brains at E13.5 (G) or E15.5 (H) and analyzed 18 h later. (I, J) Quantification of the percentage of apical processes still attached at the ventricular surface in the injected/electroporated brains at E13.5 (I) or E15.5 (J) and analyzed 18 h later. *, *p*<0.05; unpaired two-tailed *t*-test. All scale bars represent 50 µm.

To determine whether the cell positioning defects following β1 integrin blockade are due to disruption of NSC morphology, we simultaneously performed electroporation of an RFP-expressing plasmid (CAG-RFP) with the Ha2/5 antibody injection to fluorescently label a population of VZ cells at the time of β1 integrin inhibition (Figure 4). We used electroporation parameters previously shown to transfect only VZ cells [36] and determined whether cells had detached from the ventricle surface within 18 h of co-electroporation/injection (Figure 4A and 4B). To do this, sections were stained with phalloidin to label the actin ring at the border of the NSC apical membranes so that the apical processes attached at the ventricular surface could be unambiguously identified (Figure 4C–4F; further 3-D examples can be seen in Figure S5 and Video S1). Volumetric reconstructed slices were created by image analysis and both the numbers of cell soma and apical processes were counted to generate a soma∶process (S∶P) ratio (Figure 4C–4H), and the percentage of apical processes still attached at the ventricular surface was also determined (Figure 4I and 4J). There was a significant difference between the two groups (*, *p*<0.05, unpaired two-tailed *t*-test), with ITC-injected embryos having a lower S∶P ratio and a higher percentage of apical processes in contact with the ventricular surface compared to the brains injected with the β1 integrin blocking antibody at both E13.5 (Figure 4G and 4I) and E15.5 (Figure 4H and 4J). This 3-D analysis therefore identified a morphometric change in neocortical VZ cells following Ha2/5 injection, with β1 integrin blockade resulting in detachment of apical processes from the ventricular surface as shown in Figure 4A. Furthermore, we also identified many dystrophic ascending basal processes emanating from the VZ cells (Figure 4A and 4B) indicating that apical detachment has widespread morphological effects on VZ cells and may therefore adversely affect neuronal migration.

**Figure 5:**
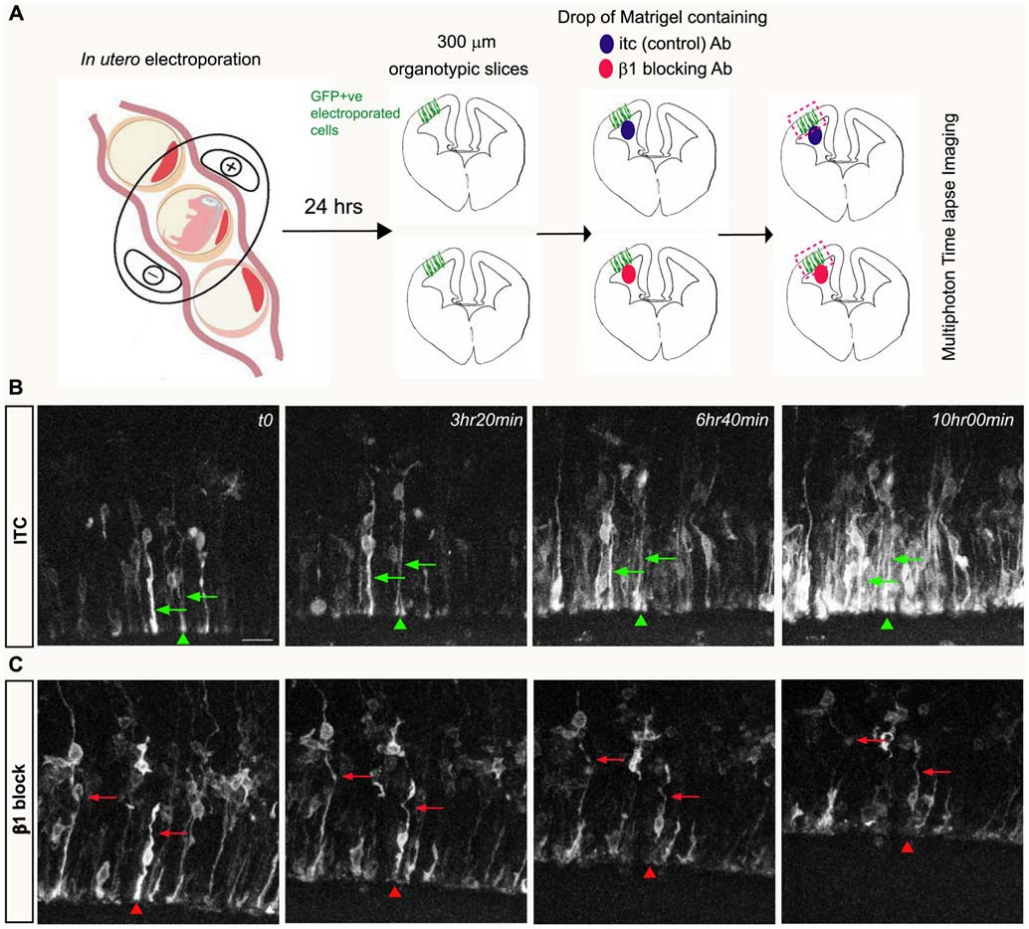
Time lapse analysis of NSC morphology and detachment after β1 integrin signalling blockade at the VZ surface. (A) Experimental paradigm of the multiphoton time lapse experiments. Organotypic brain slices were prepared 24 h after in utero electroporation with eGFP-F DNA into a wild-type E14.5 embryo. A drop of growth factor–reduced matrigel containing either ITC control or β1 blocking antibody was placed into the lateral ventricle. Automated multipoint scanning using a multiphoton laser (850 nm) was used to simultaneously monitor the behavior of two electroporated slices containing the drop of matrigel with either β1 blocking or the ITC control antibody. (B, C) Time lapse images of 10h recording of VZ neuroepithelial integrity in the presence of ITC control (B) or β1 blocking (C) antibody. (B) Slices in the presence of ITC antibody retain bipolar eGFP-F+ cells with straight processes (green arrows) and end-feet attached to the ventricular surface (green arrowheads) throughout the 10 h of recording. (C) In presence of β1 blocking antibody, the radial morphology of the eGFP-F cells is progressively disrupted; both basal and apical processes appear convoluted (red arrows) and the end-feet are detached from the ventricular surface (red arrowheads). Scale bar represents 25 µm.

To visualize the impact of β1 integrin signalling blockade on VZ cell morphology in real time, we performed time lapse imaging of neocortical VZ cell dynamics in living slices following electroporation with farnesylated enhanced green fluorescent protein (eGFP-F) (Figure 5). To specifically block β1 integrin at the apical surface without disrupting its function at the pial surface, we applied a drop of growth factor-reduced matrigel containing the antibody (β1 integrin blocking or ITC control) inside the lateral ventricle of the living slices prepared from E14.5 embryos electroporated 24 h earlier (Figure 5A). Analysis of the diffusion of β1 integrin blocking antibody-FITC from the drop of matrigel into the neocortical wall via pixel intensity profiles revealed that β1 integrin blocking antibody is mainly present in the 1/5 of the neocortical wall next to the ventricle in living slices; this corresponds to the VZ/SVZ compartment and indicates that, as with the in utero experiments, the blocking antibody does not reach the pial surface (Figure S6). This enabled us to monitor the effect of localized antibody blockade on eGFP-F+ cell morphology in the slices. After 10 h of contact with the antibody, we noted the progressive bending of both basal and apical processes (red arrows, Figure 5C and Video S3) as well as the detachment of apical end-feet from the ventricular surface (red arrowheads, Figure 5C and Video S3). In contrast, in the control experiment, NSC apical and basal processes are unaffected (Figure 5B and Video S2). Collectively, these data demonstrate that β1 integrin signalling disruption at the ventricular surface results in a progressive destabilization of the VZ architecture due to the simultaneous loss of both NSC bipolar morphology and apical end-feet at the ventricular surface.

### The Neocortex of the Laminin α2 Deficient Mouse Exhibits Three Phenotypes Similar to Those Seen Following β1 Integrin Blockade

**Figure 6:**
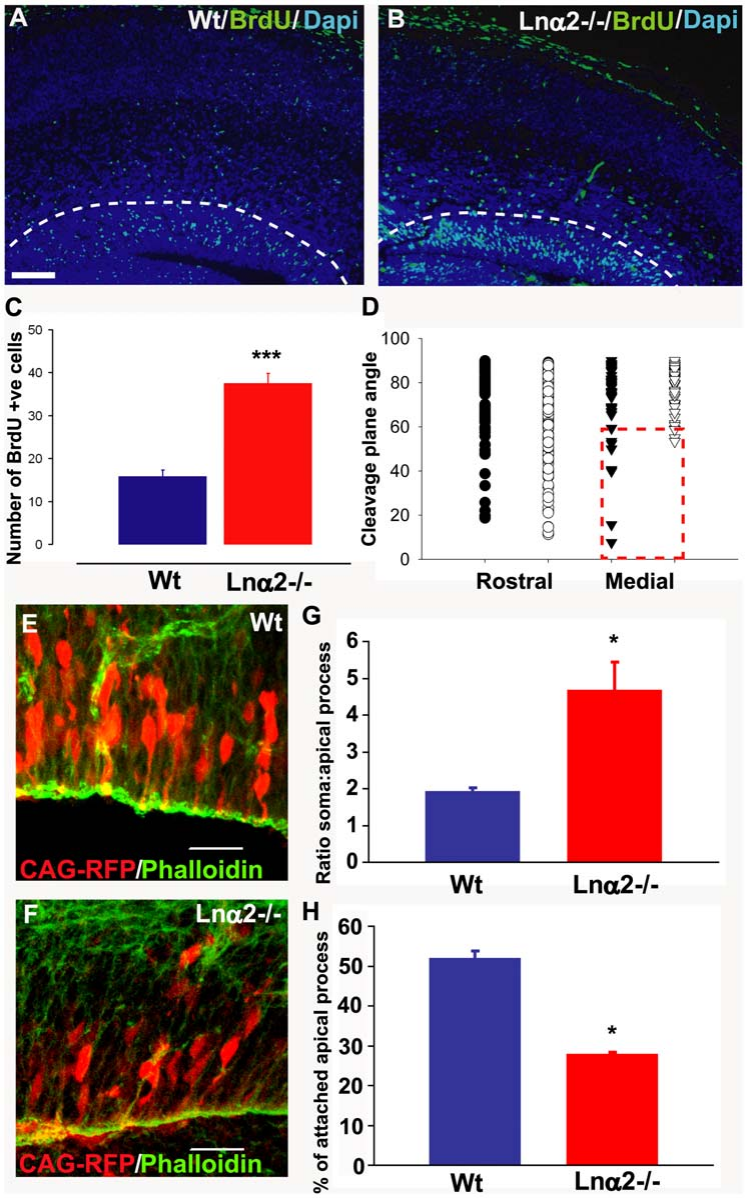
VZ cell mitotic parameters and apical process attachment are altered in Lnα2^−/−^ deficient brains. (A, B) Micrographs of dapi (blue) and BrdU (green) in E16 telencephalon of wild-type (A) and Lnα2^−/−^ (B) littermates after a 1 h BrdU pulse, scale bar, 100 µm. (C) Quantification of BrdU+ cells in the intermediate zone, outside the VZ/SVZ as marked by white dashed line in micrographs. Statistical analysis using an unpaired two-tailed *t*-test revealed a statistically significant difference ***, *p*<0.001. (D) Distribution of mitotic rostral and medial progenitors according to their cleavage angle. Boxed regions highlight the paucity of horizontal cleavages (lower than 60 degrees) in Lnα2^−/−^ embryos. *n* = 3 wild-type and four Lnα2^−/−^ embryos from one litter, ±SEM. (E, F) Confocal fluorescence micrographs showing VZ/SVZ of Lnα2^−/−^ mutant (F) or wild-type littermates (E) embryos electroporated at E15.5 with CAG-RFP (red) and stained for phalloidin (green) 18 h later. (G) Quantification of the ratio of soma to apical processes (*n* = 2 wild-type and 2 Lnα2^−/−^ embryos from one litter, ±SEM). (H) Quantification of the percentage of apical processes still attached at the ventricular surface (*n* = 2 wild-type and 2 Lnα2^−/−^ embryos from one litter, ±SEM). *, *p*<0.05; unpaired two-tailed *t*-test. Scale bars represents 100 µm (A) and 50 µm (E, F).

The laminin α2 chain (accession number Swiss Prot Q60675) mediates cell adhesion through β1 integrins [37] and is expressed at the embryonic ventricular surface [19]. Thus, laminin α2 chain-β1 integrin interactions may be involved in the NSC adhesion at the ventricular surface during corticogenesis. To test this possibility, we analyzed cell proliferation and mitotic cleavage parameters in the VZ of laminin α2 deficient mice (Lnα2^−/−^ mice) [38]. As with β1 integrin blockade, more proliferating cells were present outside the VZ/SVZ after a 1 h BrdU pulse in Lnα2^−/−^ embryos (Figure 6A–6C). Furthermore, the angle of VZ cell division was also altered in Lnα2^−/−^ embryos with the proportion of horizontal divisions (0–30 degrees) significantly lower in the medial region of the telencephalon (Figures 6D and S3B). Using the same experimental paradigms as in the β1 integrin blockade experiments, we performed in utero electroporation of the CAG-RFP plasmid in E15.5 Lnα2^−/−^ mutant embryos (Figure 6F) and control wild-type littermates (Figure 6E). We then quantified the numbers of cell soma and apical processes and determined both the S∶P ratio (Figure 6G) and the percentage of apical processes (Figure 6H) still in contact with the ventricular surface. There was a significant difference between the two groups (*, *p*<0.05, unpaired two-tailed *t*-test) with a higher S∶P ratio in the Lnα2^−/−^ mutants compared to the controls, consistent with an apical detachment of electroporated NSC. These results show that disruptions of either β1 integrin or of a ligand expressed in the VZ lead to identical alterations in cell position, NSC proliferation, orientation of cell division, and apical process detachment. These results with the Lnα2^−/−^ mice therefore identify laminin α2 as a key ligand for the integrins expressed in the VZ and thus provide a genetic corroboration of our antibody perturbation studies.

### Transient Embryonic Disruption of β1 Integrin Signalling Perturbs Postnatal Cortical Cell Layering

**Figure 7:**
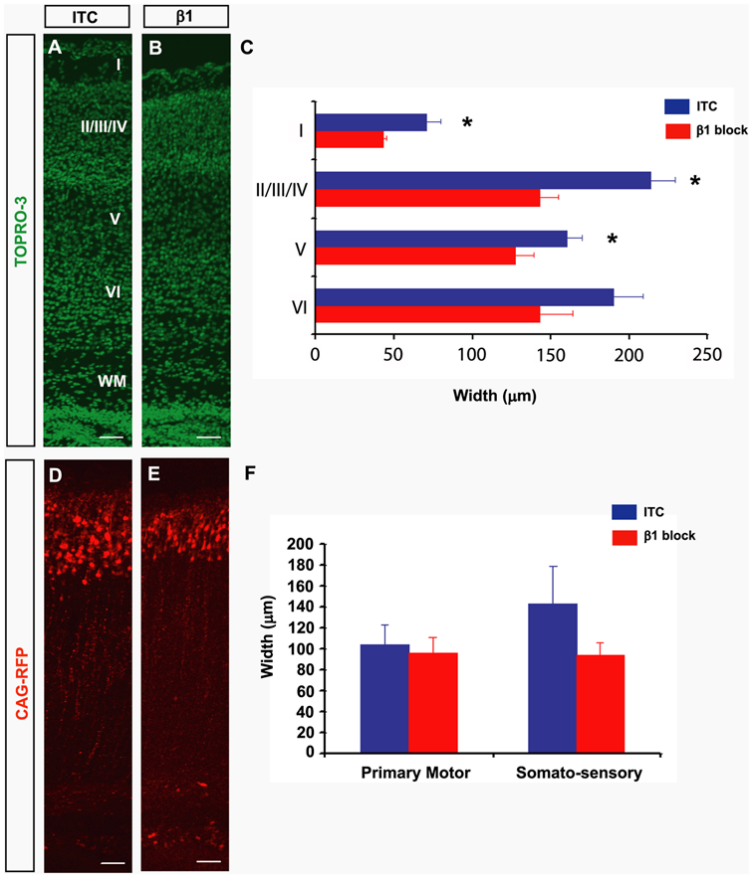
Inhibition of β1 integrin signalling at E15.5 alters cortical cell layering at P4. Fluorescence micrographs of the P4 telencephalon stained with Topro-3 after injection of the ITC (A) or β1 (B) integrin blocking antibody at E15. (C) Quantification of cortical layer thickness shows a significant reduction in the thickness of layers I–V after injection of the β1 integrin blocking antibody (layer I: ITC = 71 µm±8, β1 block = 43 µm±2, *, *p*<0.02; layers II, III, IV: ITC = 214 µm±15, β1 block = 142 µm±13, *, *p*<0.004; layer V: ITC = 160 µm±10, β1 block = 127 µm±12, *, *p*<0.04; layer VI: ITC = 190 µm±19, β1 block = 142 µm±21, *p*<0.1). Fluorescence micrographs of the P4 telencephalon illustrating CAG-RFP+ cells after injection of the ITC (D) or β1 (E) integrin blocking antibody at E15.5. (F) Quantification of the thickness of CAG-RFP+ cell layers in primary motor (PM) and somato-sensory (SS) cortices shows a reduction at the level of the somato-sensory cortex after injection of the β1 integrin blocking antibody (PM: ITC = 104 µm±18, β1 block = 95 µm±16, *p*<0.7; SS: ITC = 143 µm±35, β1 block = 93 µm±14, *p*<0.3). *n* = 2 ITC and 3 β1 integrin blocking antibody-injected embryonic brains, ±SEM. Statistical analysis was done using an unpaired two-tailed *t*-test, *, *p*<0.05. All scale bars represent 50 µm.

To investigate the long term consequences of β1 integrin blockade and detachment of VZ cells on neocortical morphogenesis and layer formation, we utilized the co-electroporation/antibody injection strategy to mark cells at E15.5 and then allowed cortical development to proceed until postnatal day (P) 4. We reasoned that VZ cell detachment may lead to disruption of cortical layering since the detached NSCs with dystrophic radial fibers that we observed in the short-term experiments would not generate the proper amount of committed neurons and would alter the migration route to the cortical plate. Indeed, we found a reduction in the width of cortical layers I-V (Figure 7A–7C), as well as in the radial distribution of RFP+ cells in the somato-sensory cortex following β1 integrin antibody injection (Figure 7D–7F). Interestingly, in keeping with the rostro-caudal differences in β1 integrin blockade described in Figure 3 spatial discrepancies were also found in the postnatal cortex of animals injected with β1 integrin blocking antibody at E15.5; cortical layer thickness was reduced in somato-sensory but not in the primary motor cortex, although these results did not reach statistical significance. These results therefore provide evidence that proper maintenance of apical process attachment during embryogenesis is critical not only for INM and NSC proliferation, but also for neuronal migration and cortical cell layer formation, as a result of which transient disruption of β1 integrin signalling can have long lasting effects.

## Discussion

### Integrins and Laminins Provide Adhesive Signals That Retain NSC within the Neocortical Niche and Maintain Its Integrity

Using a multidisciplinary approach that includes cellular/molecular analysis and multiphoton time lapse imaging, we have revealed a hitherto unsuspected role for β1 integrin during neocortical development. Previously, β1 integrin has been suggested to be important for neocortical formation through its regulation of the radial glial contacts on the pial basement membrane [20],[39]. In our study, we combined in utero electroporation and injection of a specific blocking antibody to specifically inactivate the β1 integrin receptor by preventing binding to its ligand (laminin) at the ventricular surface. Compared to transgenesis or siRNA knock down, which cause widespread effects throughout both cell and tissue, this novel approach resulted in a focused and transient disruption at the subcellular level that resulted in detachment of the apical processes of many NSC from the ventricular surface and led to increased numbers of ectopic proliferating cells as well as perturbations to INM. Confirming that integrins act at least in part through interactions with laminins in the neocortical VZ, we found similar abnormalities in the laminin α2-deficient mouse. Together, our data clearly demonstrate for the first time in vivo, to our knowledge, that integrin/laminin interactions at the apical VZ surface play a critical role in the adhesion that maintains the stem cells within their niche and preserves the architecture of the VZ.

The adhesion of stem cells to their niche is critical for the molecular programmes that promote maintenance. For example, altered expression of adhesion-related genes is known to cause depletion of haematopoietic and epidermal stem cell niches [40],[41]. A recent report has also shown that niche-supporting gonad cells in *Drosophila* also require integrin signalling to ensure niche integrity [42]. Although the VZ lacks a basal lamina, which is well recognized as a principal site of cell/extracellular matrix interactions, we have shown previously that both laminins (α2, α4, and α2 chains) and integrins are expressed at the apical surface of the neocortical wall in the embryonic mouse VZ [19]. Our present observations suggest that integrin/laminin interactions are necessary to enable the retention of apical processes seen for at least 5 h after mitosis, and which may be critical for key cell-cell interactions that instruct behaviour [6]. So, while no perturbation of cell differentiation following β1 integrin blockade has been detected in our study, premature loss of these interactions resulting from apical process detachment has profound consequences on other aspects of NSC behaviour, including dysregulated proliferation of the NSC and altered allocation to the developing cortical plate. Interestingly a recent investigation of the role of α6β1 integrin in the adhesion of adult SVZ progenitor cells to endothelial cells using the same in vivo blocking antibody paradigm demonstrated two similar phenotypes to those we observed in the embryo—separation of SVZ progenitor cells from their normal location (adjacent to blood vessels) and enhanced proliferation [43]. Integrin/laminin interactions may therefore play similar roles in the regulation of neural stem and progenitor behaviour in embryonic and adult central nervous system. Whether blood vessels in the embryonic NSC niche provide some of these laminins as they do in the adult remains unknown, but recent studies do suggest a critical role of the developing cortical vasculature in regulating cortical neurogenesis [44],[45] and laminin α2 is expressed in blood vessels in the embryonic VZ [19]. Further work to test the hypothesis that laminin/integrin interactions in the vicinity of blood vessels contribute to the embryonic niche as they do in the adult is therefore required.

### The Laminin/Integrin Interaction Is Also Necessary for Proper INM and the Orientation of NSC Division

**Figure 8:**
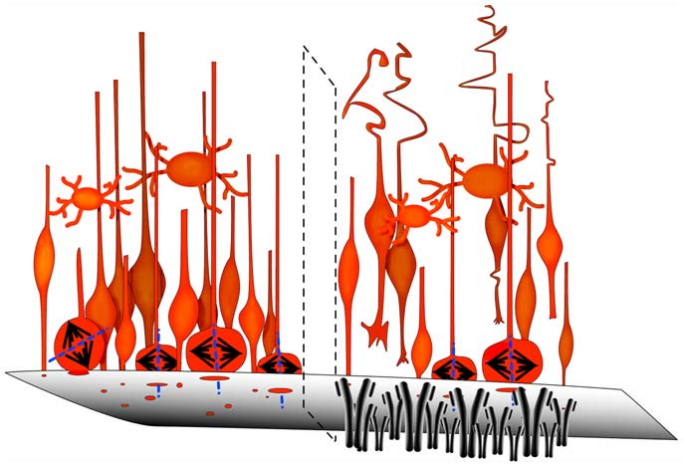
Model depicting the role of β1 integrin in the VZ. Schematic (left) showing the normal VZ with NSC attached at the ventricular surface undergoing mitosis in a variety of orientations. After β1 integrin blocking antibody injection (right), NSC detach from the ventricular surface and horizontal cell divisions are no longer present at the ventricular surface.

The two other phenotypes we observed after disruption of integrin signalling in the VZ, the loss of the subset of VZ cells that divide with horizontal cleavage planes and abnormal cortical layer formation may not simply be explained by an effect solely on VZ adhesion. Under normal circumstances, horizontal cleavages are the minority, and daughter cell fate cannot be predicted solely by the cell division orientation of its parent cell [31],[46],[47], because cells undergoing vertical cleavage during mitosis can give rise to either identical (via symmetric division) or different (via asymmetric division) daughter cells [48]. However, several recent studies have suggested an important link between the precise regulation of mitotic spindle orientation and the fate of neocortical neural progenitors. In particular, disruptions of centrosomal proteins such as Aspm [33], Nde1 [49], doublecortin-like kinase [50], and Cep120 [51], all of which play crucial roles in mitotic spindle function, affect the neural progenitor pool size and lead both to alterations in INM [51] and reductions in cerebral cortical size [49]. Although the link between mitotic spindle orientation and daughter cell fate is still debated, several recent studies demonstrate the close relationship between VZ cell cleavage angle and the location of the resulting daughter cells (i.e., ventricular surface versus abventricular location) [46],[48; present study]. While the detailed molecular mechanisms by which the integrin/laminin interaction influences the NSC cleavage orientation are still not known, compelling data linking integrin signalling to spindle assembly have already been reported for Chinese Hamster Ovary cytokinesis in vitro [34]. Thus our present results extend the importance of this role by demonstrating that β1 integrin signalling is required for the regulation of NSC mitotic spindle dynamics for the cells that normally undergo oblique cleavages during neocortical neurogenesis in vivo (Figure 8). Furthermore, our data suggest regional differences in that medial and caudal telencephalic progenitors are most sensitive to β1 integrin signalling. In keeping with this, it is interesting to note that human congenital muscular dystrophy caused by deficiency of the laminin α2 chain has been associated with significant abnormalities of cortical development in the occipital but not frontal regions of the telencephalon [52].

### Inhibiting the Laminin/Integrin Interaction Leads to Impaired Corticogenesis

The thinning of the cortical layers that we observed in the postnatal mouse brain following transient blockade of integrin signalling in the embryo might reflect the alterations in the plane of cell division and subsequent effects on neurogenesis, but a key observation argues against this. We found that the NSC proliferation and morphological defects occurring after β1 integrin blockade had long-term consequences on the migration of both the newly formed neurons as well as those previously generated before the antibody injection. For example, the deep cortical layers (IV and V), which contain neurons born before the perturbation on E15.5, were also thinner than in controls. This deep layer defect is not likely to be caused solely by a disruption in neurogenesis at midgestation, since the earlier born neurons would be expected to establish proper laminar positions. Rather, it points to a phenotype resulting from the dystrophic radial glia processes we observed in the antibody-injected tissue, with cells born several days prior to the injection and still en route to the cortical plate affected by the morphological changes to the radial glia in the VZ. The most parsimonious explanation is that the loss of apical adhesion leads to NSC detachment and shortening and dystrophy of the basal process, and this in turn perturbs the migration of the cells on these processes. Supporting this proposed mechanism, several reports have demonstrated that mechanical forces play an important role in shaping the developing brain [7],[53],[54]. The present data suggest that anchorage of the apical endfeet provides the physical tension required for maintenance of position and morphology of radial glia cells during corticogenesis. Thus, our data using short-term blocking approaches reveal functions not shown by knock-out experiments and clearly define the novel contribution of integrins to neocortical development by elucidating a number of key roles in the regulation of NSC behaviour in the mammalian VZ.

## Materials and Methods

### Animals

Control ICR mice were produced in the Children's National Medical Center (CNMC) animal facility. ICR (CNMC), C57/BL6 (National Institute on Aging, NIA), and laminin α2 deficient mice (Oregon Health and Science University, OHSU and also SUNY Stony Brook, NY) were housed under standard conditions with access to water and food ad libitum on a normal 12 h light/dark cycle. Genotyping for the laminin α2 deficient mice was performed as previously described [38].

### Tissue Sectioning

6- or 20-µm thick coronal sections from frozen Tissue-Tek embedded embryonic brains were harvested from three different levels along the rostro-caudal axis depending on the experiment. Most of the studies focused on the medial region of the forebrain, corresponding to E13/E14 plate 4 (for E13 to E14 embryos) or E15/E16 plate 5 (for E15 to E16 embryos) in the atlas by Jacobowitz and Abbott (1998). The analysis of the cleavage plane angle was also performed on the rostral (E13/E14 plate 3; E15/E16 plate 3) and caudal (E13/E14 plate 5; E15/E16 plate 8) regions of the embryonic forebrain [55].

### Intraventricular Injection/Electroporation

Intraventricular injections were done with approval from the NIA and CNMC Institutional Animal Care and Use Committees using methods described previously [36]. Briefly, timed-pregnant mice (from E12 to E15) were anesthetized with ketamine/xylazine and a midline laparotomy was performed exposing uterine horns. The lateral ventricle in the brain of each embryo was visualized with transillumination and the injections were performed with a glass capillary pipette (75–125 µm outer diameter with bevelled tip) driven either by a Sutter micromanipulator (Sutter Instrument Company) equipped with 20-µl Hamilton gas-tight syringe or a nitrogen-fed Microinjector (Harvard Apparatus). For integrin blocking studies, approximately 1 µl of either β1 integrin blocking antibody (10 ng or 100 ng; Ha2/5, with or without FITC conjugation, BD Pharmigen) or an ITC antibody (anti-hamster, BD Pharmigen) solution (combined 3∶1 with sterile fast green dye to enable monitoring of the injection into the cerebral ventricles, Sigma) was injected alone or mixed with DNA. Two different plasmid vectors were used: a plasmid encoding red fluorescent protein under the control of the chicken β actin promoter (CAG-RFP) and a plasmid expressing eGFP-F (Clontech). For the in utero electroporation procedure, the anode of a Tweezertrodes (Genetronics) was placed above the dorsal telencephalon and four 40-V pulses of 50 ms duration were conducted across the uterine sac. Following intrauterine surgery, the incision site was closed with sutures (4-0, Ethicon,) and the mouse was allowed to recover in a clean cage. Mice were humanely killed 8–24 h after the injection unless indicated otherwise and embryonic brains were harvested.

### Slice Culture and Multiphoton Time Lapse Imaging

Organotypic slices were prepared 24 h after in utero electroporation performed on E14.5 brains with an eGFP-F plasmid as described previously [30],[56]. Briefly, 300-µm-thick slices containing EGFP-F+ cells were collected in ice-cold Complete Hank's Balanced Salt Solution using a vibrating microtome (Leica VT1000S) and transferred into serum-free medium (SFM; neurobasal medium supplemented with B27, N2 and glutamax; Invitrogen). After 1 h of recovery, the slices were placed in a 35-mm glass bottom culture dishes. ITC control or β1 integrin blocking antibodies were diluted (1∶100) in growth factor-reduced matrigel (BD Biosciences) and a drop (0.5 µl) of this solution carefully introduced in the ventricular space of the embryonic brain slices. A slice holder immobilized the slices and 3 ml of SFM were added. The 35-mm glass bottom culture dishes containing the slices with matrigel were positioned in a heated micro-incubation chamber (DH-40i; Warner Instruments). Preheated SFM was pumped over the slices for the length of the imaging experiment (usually 10 h), the slice temperature was maintained at 37°C and the imaging preparation was maintained in 5% CO_2_/95% air for the entire period. All multiphoton imaging was performed on a Zeiss LSM 510 Meta NLO system equipped with an Axiovert 200M microscope (Zeiss) direct coupled to a Mira 900F laser pumped by an 8-W Verdi laser (Coherent Laser Group). EGFP was excited at 850 nm and time-series experiments were conducted under oil-immersion with 25× objective. Time-series images consisted of 40-µm-thick z-stacks and were collected at multiple locations at 2 min intervals to repetitively record both β1 integrin blockade and control slices. The experiments were analyzed with LSM 510 software. For the presentation of videos, each z-stack was projected onto one optical slice per time period and the resulting frames were assembled and compressed using Volocity software (Improvision).

For analysis of the diffusion of the blocking antibody in the experiments using matrigel to deliver antibodies within organotypic slices, these slices were prepared as above. After 10 h of incubation with the ITC control or FITC-labelled β1 integrin blocking antibodies in a drop of growth factor-reduced matrigel placed in the ventricular cavity, slices were fixed in 4% PFA and nuclei stained with dapi. 20-µm-thick z-stacks were collected and were analyzed with LSM 510 software. Each neocortical length was divided in five bins, each representing 20% of the total cortical thickness. The pixel intensity calculated by the LSM software was summed for each bin and then averaged and plotted in a graph (Figure S6).

### Image Analysis

20-µm-thick coronal sections were imaged using a Zeiss LSM 510 NLO system direct coupled to an inverted Axiovert 200M microscope (Zeiss). 25× (DIC, 0.8 na; Zeiss) image stacks (1-µm intervals) containing the region/cells of interest were collected with conventional detectors and then analyzed post hoc. For the orientation of cell division, studies were conducted with a 40× oil-immersion lens (DIC, Plan Neofluar, 1.3 na; Zeiss). Each frame of the series, consisting of a z-stack of images, was reconstructed in 3-D using Zeiss LSM software and was then rotated around the *y*-axis to bring the edge of the mitotic figures at the VZ surface into view so that the mitotic spindle plane was parallel to the computer screen. The angle of the mitotic spindle was then measured by projecting a line through the spindle to a reference line parallel with the ventricular surface. This procedure was repeated for each mitotic figure in each frame from the beginning of metaphase (a discrete organized metaphase plate) until the beginning of chromatid separation in anaphase. The spindle angles were then documented manually and graphed using SigmaPlot software.

The 3-D reconstruction of CAG-RFP cell attachment to the ventricular surface labelled with phalloidin was performed using Volocity software (Improvision).

### Western Blot Analysis

For integrin signalling validation, half the litter was injected with the blocking antibody and the other half with the control antibody for 30 min. Telencephali were isolated by rapid dissection 30 min after injection and then flash frozen. Total brain lysates were prepared by resuspending the tissue in cell lysis buffer. Tissue protein was extracted using T-PER tissue protein extraction buffer with protease inhibitor cocktail (Sigma) and protein concentration was determined by the BCA protein assay kit (Pierce). 50 µg of protein was separated by SDS-PAGE (8%–12%) and transferred to nitrocellulose membranes. The membranes were blocked in 5% nonfat milk for 1 h at room temperature, followed by an overnight incubation at 4°C with antibodies raised against p-Akt1 (BD Pharmigen), total Akt (T-AKT, BD Pharmigen), or β-actin (Sigma). Membranes were then washed and incubated with secondary antibodies for 1 h at room temperature. Protein bands were visualized using a chemiluminescence detection kit (Amersham Biosciences).

### Immunohistochemistry and Staining

Embryonic brains were fixed in 4% paraformaldehyde (PFA) in PBS overnight at 4°C before being transferred to sequential 20% and 30% solutions of sucrose (w/v) and left at 4°C overnight or until the brains equilibrated. The brains were then embedded in TissueTek (Sakura) prior to cryostat sectioning (Leica CM3050S). For immunofluorescence, sections were blocked for a minimum of 30 min in PBS containing 0.1% Triton X-100 and 10% normal goat serum (Sigma). Sections were incubated overnight with primary antibodies at 4°C. After incubation with the appropriate secondary antibodies and counter-staining with 4′,6-diamidino-2-phenylindole dihydrochloride (Dapi, Sigma) to visualize the DNA, images were acquired using an Olympus IX50 fluorescence microscope. Images were processed using MagnaFire and Photoshop 6.0 (Adobe) and adjusted such that the entire signal was in the dynamic range. The following antibodies were used for immunofluorescence: anti-β1 integrin (used 1∶100 in blocking buffer), anti-Tbr2 (used 1∶200 in blocking buffer following a 5 min boil in 10 mM sodium citrate, Millipore), anti-RC2 (used 1∶5 in blocking buffer, Developmental Studies Hybridoma Bank), anti-phospho histone H3 (used 1∶500 in blocking buffer, Millipore), anti-BrdU (used 1∶5 in BrdU blocking buffer, Accurate Chemicals), anti-β3 tubulin (used 1∶500 in blocking buffer, Sigma). For BrdU staining, the blocking buffer consisted of DMEM (Sigma) supplemented with 1% tween-20 (Sigma) and 7 mg of DNAse (Sigma) per 1 ml of blocking solution.

The angle of the cleavage plane was determined in cells in anaphase identified by propidium iodide staining performed on 20-µm-thick sections from three different levels (rostral, medial, and caudal) of the forebrain from E13 to E16 embryos. F-actin filaments were visualized at the ventricular surface using Alexa Fluor 488 phalloidin (165 nM final concentration, Molecular Probes) by incubation for 1 h after a prior 5 min incubation in 0.1% Triton X-100 in PBS and 30 min in 10% normal goat serum in PBS for blocking. Nuclear counterstaining was performed by 10 min incubation at room temperature in TOPRO-3 iodide (1∶100, Molecular Probes).

For the subcellular localization analysis of β1 integrin, first a postfixation with methanol at −20°C for 10 min was performed on the cryosections before a blocking step in a solution containing bovine serum albumin 3% and Tween 0.05% for 1 h at room temperature (RT). Then, anti-β1-integrin antibody (clone MB1.2 from Chemicon Int., 1/100 dilution) and Alexa Fluor 546 coupled phalloidin (Molecular Probes, 1/200 dilution) were incubated overnight in the blocking buffer at RT. Alexa Fluor 488-conjugated donkey anti-rat antibody (Molecular probes, 1/250 dilution) was incubated for 1 h at RT along with Hoechst for nuclei staining. Images were captured with a Zeiss confocal using an oil immersion 63× objective with a zoom of 2. The profile function of the Zeiss acquisition software was used to determine the fluorescence intensity of each marker at a defined *xy* position.

### Quantitative Analysis

For PH3 analysis performed 18 h following antibody (ITC or β1 blocking) injection at E12.5, positive cells were calculated from the average of three sections from five separate embryos from two litters. For E15.5 injected embryos, positive cells were calculated from the average of three sections from 11 (ITC) or 17 (β1 integrin blocking antibody) separate embryos from nine litters. For BrdU analysis, labelling index was calculated on the basis of three sections from three embryos from 1 litter for both E12.5 and E15.5 injected embryos. For Tbr2 expression analysis, a 200 µm×100 µm (width×height) region adjacent to the ventricular surface was analyzed and an average was calculated on the basis of three sections from three embryos from one litter. All statistical analysis was performed using GraphPad Prism version 4.00 for Windows, GraphPad Software (www.graphpad.com). The specific statistical test is indicated in both the text and figure legends.

Apical process quantification was performed on 150-µm vibrating microtome-cut coronal sections of co-antibody injected/CAG-RFP electroporated E13.5 and E15.5 brains stained with phalloidin to label the ventricle surface. 80-µm z-stacks were collected in 2-µm steps with a 1,024×1,024 pixel frame size and each z-stack was analyzed with LSM examiner (Zeiss) and Volocity software (Improvision) to determine the number of RFP+ cell bodies within 200 µm from the ventricle surface and to determine the number of apical processes attached to the ventricle (colocalized with the phalloidin staining). Both of these counts were calculated from the average of three embryos for each condition. The ratio soma/apical processes (S∶P) representing the total number of cell bodies divided by the number of apical processes was determined and the results were analyzed by unpaired two-tailed *t*-test.

The postnatal phenotype of embryos co-injected/electroporated at E15.5 was assessed at P4 by determining the thickness of the individual cortical layers (I, II/III/IV, V, VI as shown in Figure 7A) and by the radial distribution (i.e., layer specification) of RFP+ cells in multiple areas at both the primary motor and somato-sensory cortical levels. The data from two ITC and three β1 integrin blocking antibody injected animals were analysed by unpaired two-tailed *t*-test.

### Statistical Modelling

In preparation of performing statistical analysis we checked assumptions of normality and homogeneity of variance, and found that the data for the orientation of cell division were not normally distributed and could not be transformed to achieve acceptable levels of normality to permit linear regression analysis. Thus, an ordinal logistic regression model was developed to estimate the tendency toward having greater angles of cleavage in one group (β1 integrin blocking antibody injected brains or Lnα2^−/−^ brains) compared to another (controls: ITC injected brains or wild type littermates from Lnα2^−/−^ embryos). To perform these analyses, angles of cleavage were stratified as, <30 degrees, 30 to <60 degrees, and 60–90 degrees. The model, which included covariates to account for brain region and study group, enabled the study to estimate and compare differences in the frequency of angle of cleavage strata in one study group compared to another. The model adjusted variance estimates to account for the correlation between repeated measurements on the same embryo.

## Supporting Information

Figure S1 **β1 integrin is localized basally to adherens junctions.** (A) Colabelling of β1 integrin and actin in E14 neocortical VZ reveals the basal localization of β1 integrin staining relative to actin-based adherens junctions. The white arrow points to the apical process (identified by the accumulation of actin staining at the tip) along which the fluorescence intensity profile presented in the graph below (B) was determined for each marker. To facilitate the comparison of β1 integrin positioning relative to actin, dotted lines were drawn at the peak of staining intensity for each marker and the actin position taken as the position of reference. (B) The graph shows that β1 integrin is located more basally than actin-based adherens junctions.
(8.92 MB TIF)

Figure S2 **In utero intraventricular injection of β1 integrin blocking antibody results in specific targeting of the ventricular surface and decreased β1 integrin signalling in the VZ.** (A, B) Fluorescence micrographs of the E14 telencephalon following an intraventricular injection of a β1 integrin FITC-conjugated blocking antibody (green) show that the antibody does not penetrate as far as the pial surface (white dashed line) but is present in the VZ (B) (negative control [PBS], A), (dapi-counterstained nuclei in blue). (C) Western blot analysis showing levels of phospho (p) and total (T) Akt 1 and actin in E12.5 and E15.5 embryos 30 min after injection with an ITC or β1 integrin blocking antibody.
(8.58 MB TIF)

Figure S3 **Both β1 blocking antibody-injected and laminin α2-deficient forebrains exhibit a lower proportion of horizontal mitotic cleavages in the VZ throughout neurogenesis.** (A) Graph illustrating the results of the ordinal regression analysis of the frequency of cleavage plane angle strata in the β1 integrin blocking antibody injected forebrain versus ITC by region (see Materials and Methods). Note the proportion of horizontally dividing VZ cells (0–30 degrees) is lower at the medial and caudal levels of β1 integrin blocking antibody injected forebrain compared to controls. (B) Graph illustrating results of the ordinal regression analysis of the frequency of cleavage plane angle strata in Lnα2^−/−^ forebrain versus wild type by region. Note the proportion of horizontally dividing VZ cells is lower at the medial level of Lnα2^−/−^ forebrain compared to wild-type littermates, as with the embryos injected with β1 integrin blocking antibody. *n* = 3 wild-type and 4 Lnα2^−/−^ embryos from one litter, ±SEM (standard error of the mean).
(8.04 MB TIF)

Figure S4 **Cell differentiation is not affected after disruption of β1 integrin signalling at the ventricular surface.** (A, B) Fluorescence micrographs of Tbr2 expression (green) after ITC (control, A) or β1 integrin blocking (B) antibody injection at E12.5. (C, D) Quantification of Tbr2+ cells in a dorsal (C) and ventral (D) 200 µm×100 µm (width×height) area of the VZ shows that the number of intermediate progenitors is not modified by the blockade of β1 integrin in the VZ. (E, F) Fluorescence micrographs of β3 tubulin expression (red, dapi-counterstained nuclei in blue) after antibody injection at E12.5. Note the lack of β3 tubulin expression within 100 µm of the ventricular surface; scale bar represents 50 µm.
(7.92 MB TIF)

Figure S5 **Intraventricular co-electroporation/injection of β1 integrin blocking antibody reveals detached cells.** (A) Schematic representation of the delamination analysis including orthogonal sections to view the cortex from apical to basal surface followed by volumetric reconstruction and counting of the number of apical processes and cell bodies. (B) 3-D reconstruction pictures of CAG-RFP electroporated cells (in red) at the VZ surface (labelled with phalloidin in green) of E15.5 β1 integrin blocking antibody-injected brain. (C) Confocal fluorescence micrographs of E16 telencephalon stained with PH3 (C2, green) following β1 integrin blocking antibody and electroporation with CAG-RFP (C1, red) at E15.5. Note the co-expression of CAG-RFP and PH3 in a cell (yellow, C3, C4) away from the ventricular surface. Scale bar represents 50 µm.
(10.08 MB TIF)

Figure S6 **Analysis of the mobility/diffusion of the β1 integrin blocking antibody in growth factor-reduced matrigel during time lapse imaging experiments.** (A, B) Fluorescent micrographs of 300-µm-thick neocortical E14.5 slices stained with dapi (blue) and containing a drop of ITC-(A) or FITC- conjugated β1 integrin blocking antibody (B) growth factor-reduced matrigel (GFRM) within their ventricular space. (C ,D) Intensity profile graphs illustrating the distribution of pixel intensity on both blue (dapi) and green (FITC antibody) channels from the VZ surface (bin 0) towards the pial surface (bin 5) with both ITC- (C) and β1 integrin blocking antibody FITC-conjugated (D) GFRM. Each bin represents 20% of the neocortical wall. Note that a green peak (green arrow) is only detected in the first bin of the β1 integrin blocking antibody FITC-conjugated GFRM (D), whereas a blue peak (blue arrows) corresponding to dapi at the apical surface of the neocortical slices is detected in both conditions. (E) Graph illustrating the average of green channel pixel intensity summed for each bin along the neocortical wall in β1 integrin blocking antibody FITC-conjugated GFRM (red line) compared to controls (blue line). Note that the pixel intensity in the first bin (corresponding at the apical surface) is significantly higher with the β1 integrin blocking antibody than in the control confirming that the antibody can diffuse into at least in the first 20% of the thickness of the neocortical wall. Conversely, the levels of antibody are not different from the control (noise/background) in bin 5 (that corresponds to the pial surface) indicating that the β1 integrin blocking antibody does not reach the pial surface. *n* = 6 cortices for ITC; *n* = 10 for β1 blocking antibody, ±SEM, *, *p*<0.05; unpaired two-tailed *t*-test. Scale bar represent 50 µm.
(8.14 MB TIF)

Video S1 **Interactive Quick Time VR video of CAG-RFP electroporated cells (in red) at the E16 VZ surface labelled with phalloidin (in green) following simultaneous β1 integrin blocking antibody injection and CAG-RFP electroporation at E15.5.**
(4.50 MB MPG)

Video S2 **Time lapse imaging of organotypic brain slice prepared 24 h after in utero electroporation with eGFP-F DNA into a wild-type E14.5 embryo and incubated in the presence of a drop of ITC antibody-containing growth factor–reduced matrigel in the lateral ventricular space.** Automated multipoint scanning using a multiphoton laser (850 nm) was used to monitor the behavior of the eGFP-F+ cells in presence of ITC control antibody. During the 10 h recording the bipolar eGFP-F+ cells maintain straight processes and end-feet attached to the ventricular surface. Scale bar represents 25 µm.
(1.50 MB MPG)

Video S3 **Time lapse imaging of organotypic brain slice prepared 24 h after in utero electroporation with eGFP-F DNA into a wild-type E14.5 embryo and incubated in the presence of a drop of β1 integrin blocking antibody-containing growth factor–reduced matrigel in the lateral ventricle.** Automated multipoint scanning using a multiphoton laser (850 nm) was used to monitor the behavior of the eGFP-F+ cells. In presence of β1 blocking antibody, the neuroepithelial organization of the VZ appears to be progressively disrupted because of an alteration of the radial morphology of the NSC cells whose both basal and apical processes appear bowed and exhibit detachment of their ventricular end feet. Scale bar represents 25 µm.
(1.04 MB MPG)

## Abbreviations

BrdU: 5-bromo-2-deoxyuridine
eGFP-F: farnesylated enhanced green fluorescent protein
INM: interkinetic nuclear migration
ITC: isotype control
NSC: neural stem cell
p-Akt 1: phospho-Akt 1
PH3: phospho histone 3
SVZ: subventricular zone
Tbr2: T-brain 2
VZ: ventricular zone
