# Supplementary figures and images for: β1 Integrin Maintains Integrity of the Embryonic Neocortical Stem Cell Niche
Source: PLoS Biol. 2009 Aug 18;7(8):e1000176. doi: 10.1371/journal.pbio.1000176 (PMC2720642; doi:10.1371/journal.pbio.1000176)

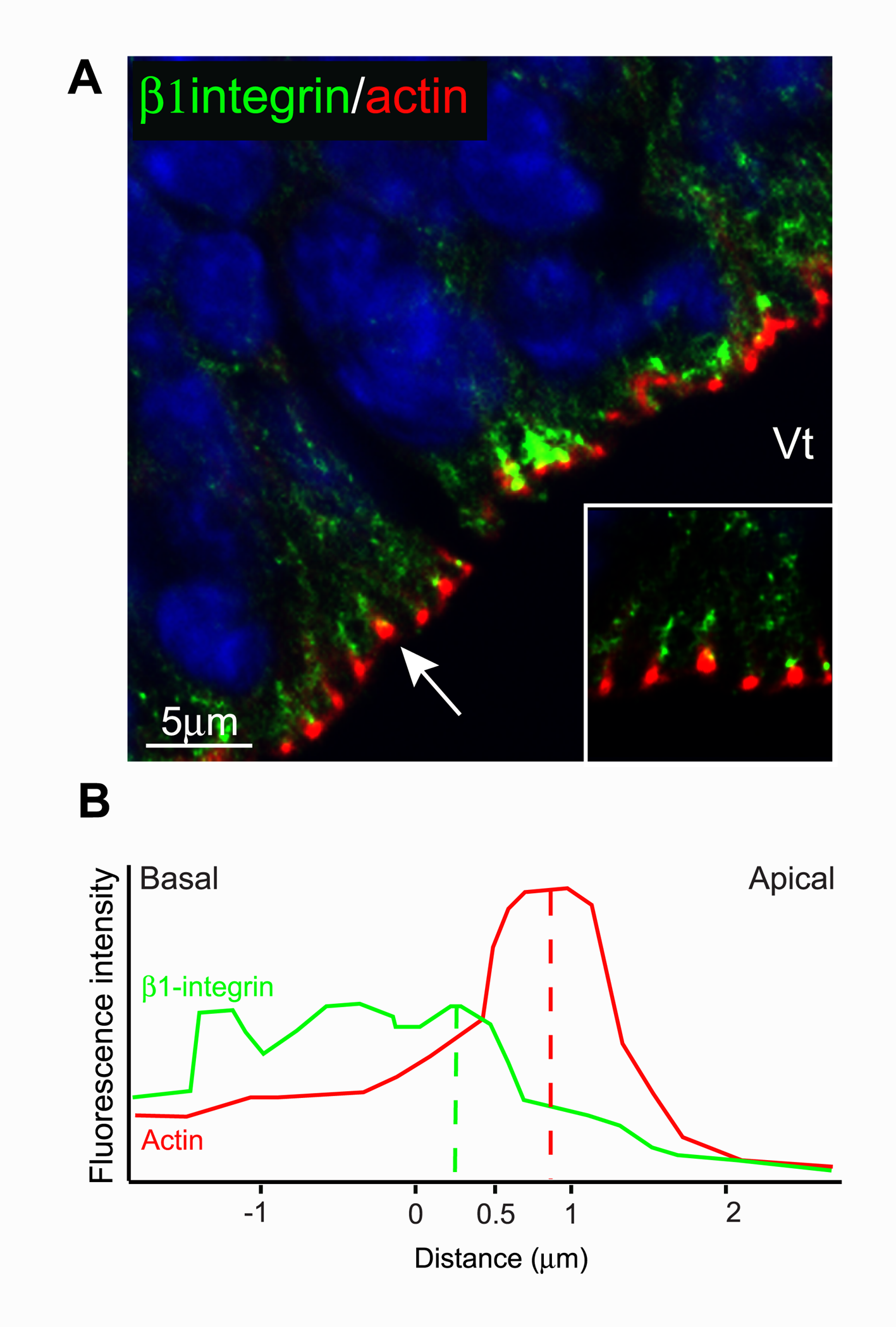

Supplement: Figure S1 — β1 integrin is localized basally to adherens junctions. (A) Colabelling of β1 integrin and actin in E14 neocortical VZ reveals the basal localization of β1 integrin staining relative to actin-based adherens junctions. The white arrow points to the apical process (identified by the accumulation of actin staining at the tip) along which the fluorescence intensity profile presented in the graph below (B) was determined for each marker. To facilitate the comparison of β1 integrin positioning relative to actin, dotted lines were drawn at the peak of staining intensity for each marker and the actin position taken as the position of reference. (B) The graph shows that β1 integrin is located more basally than actin-based adherens junctions. (8.92 MB TIF) [file pbio.1000176.s001.tif]

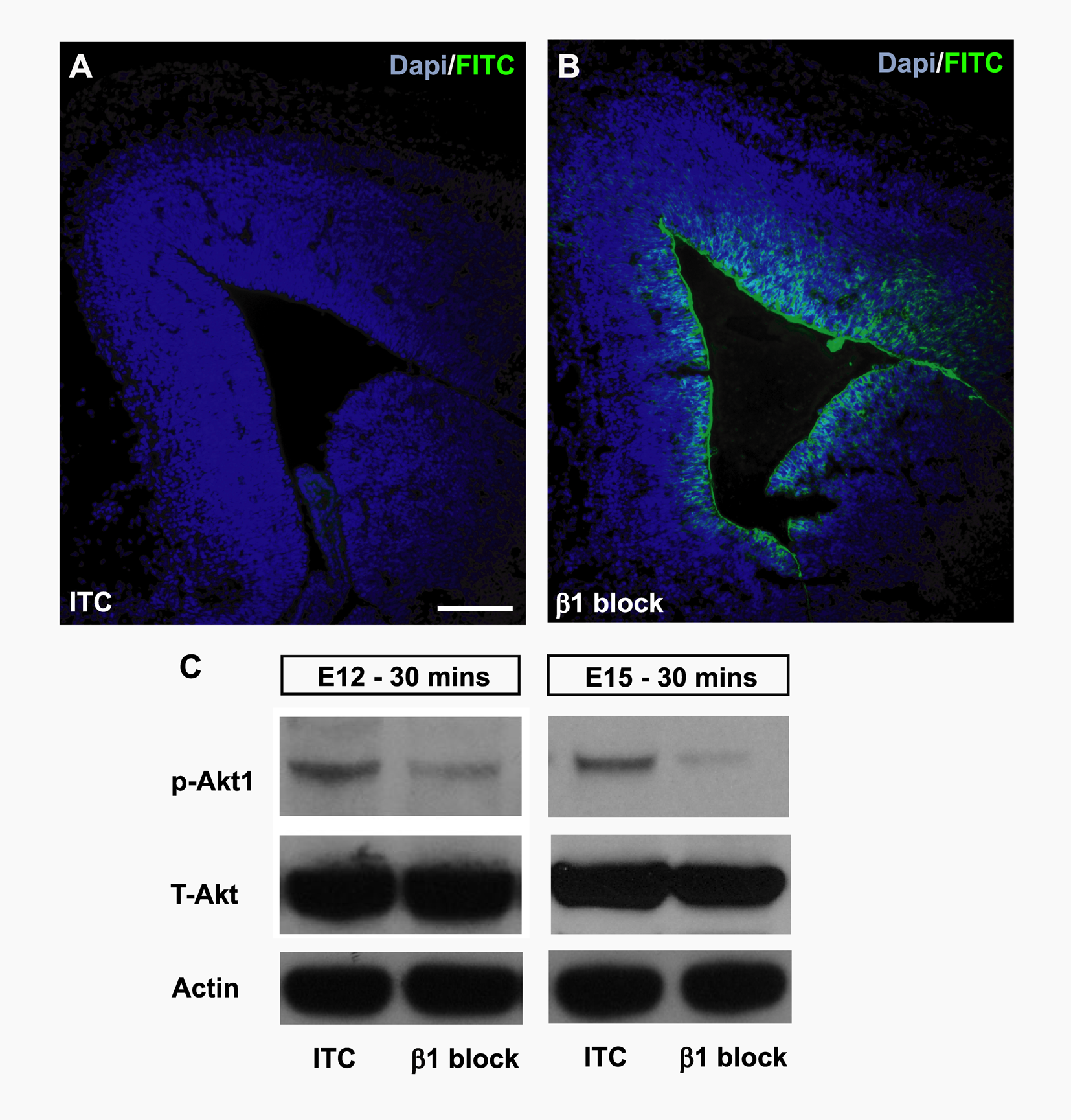

Supplement: Figure S2 — In utero intraventricular injection of β1 integrin blocking antibody results in specific targeting of the ventricular surface and decreased β1 integrin signalling in the VZ. (A, B) Fluorescence micrographs of the E14 telencephalon following an intraventricular injection of a β1 integrin FITC-conjugated blocking antibody (green) show that the antibody does not penetrate as far as the pial surface (white dashed line) but is present in the VZ (B) (negative control [PBS], A), (dapi-counterstained nuclei in blue). (C) Western blot analysis showing levels of phospho (p) and total (T) Akt 1 and actin in E12.5 and E15.5 embryos 30 min after injection with an ITC or β1 integrin blocking antibody. (8.58 MB TIF) [file pbio.1000176.s002.tif]

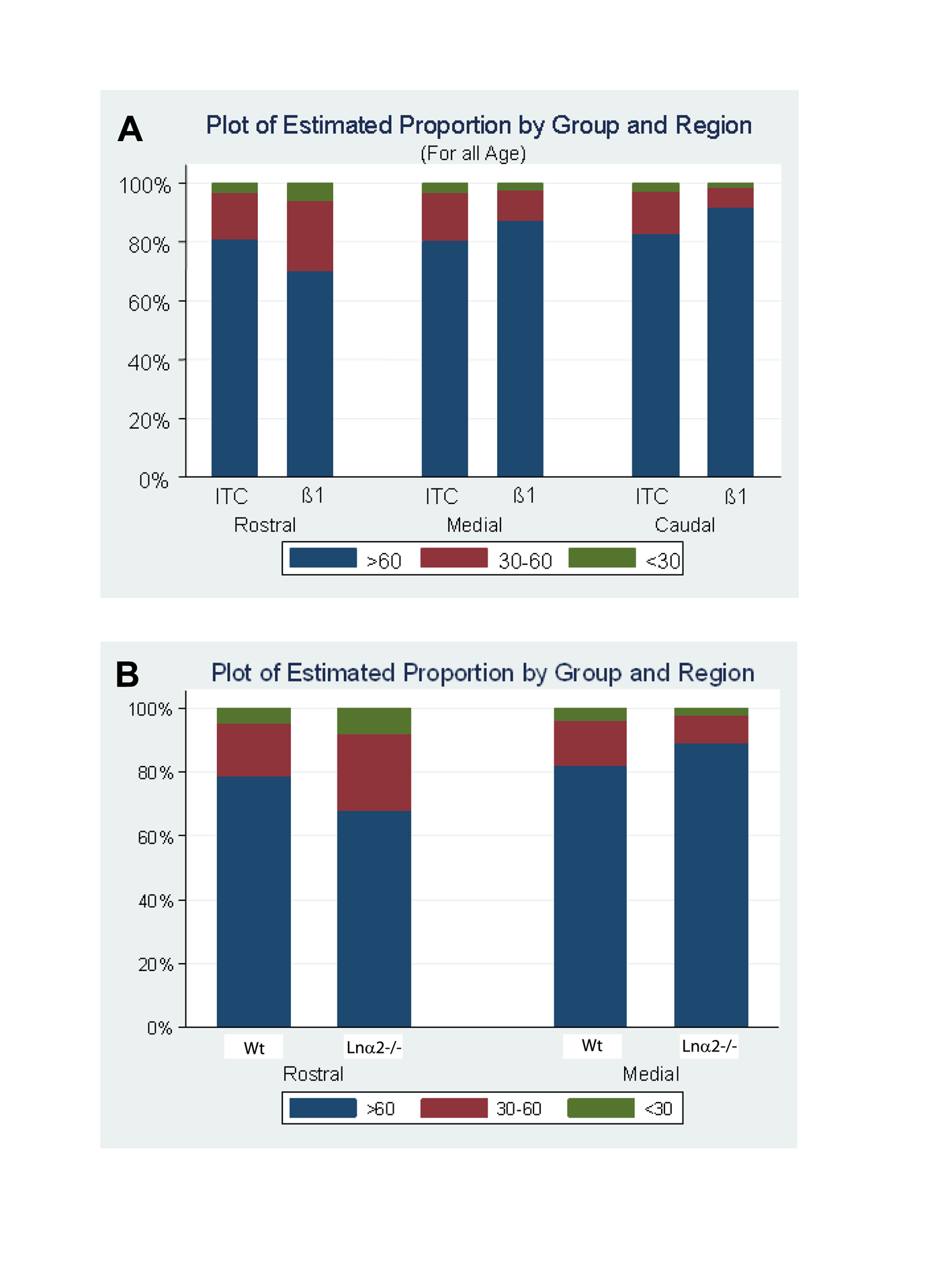

Supplement: Figure S3 — Both β1 blocking antibody-injected and laminin α2-deficient forebrains exhibit a lower proportion of horizontal mitotic cleavages in the VZ throughout neurogenesis. (A) Graph illustrating the results of the ordinal regression analysis of the frequency of cleavage plane angle strata in the β1 integrin blocking antibody injected forebrain versus ITC by region (see Materials and Methods). Note the proportion of horizontally dividing VZ cells (0–30 degrees) is lower at the medial and caudal levels of β1 integrin blocking antibody injected forebrain compared to controls. (B) Graph illustrating results of the ordinal regression analysis of the frequency of cleavage plane angle strata in Lnα2−/− forebrain versus wild type by region. Note the proportion of horizontally dividing VZ cells is lower at the medial level of Lnα2−/− forebrain compared to wild-type littermates, as with the embryos injected with β1 integrin blocking antibody. n = 3 wild-type and 4 Lnα2−/− embryos from one litter, ±SEM (standard error of the mean). (8.04 MB TIF) [file pbio.1000176.s003.tif]

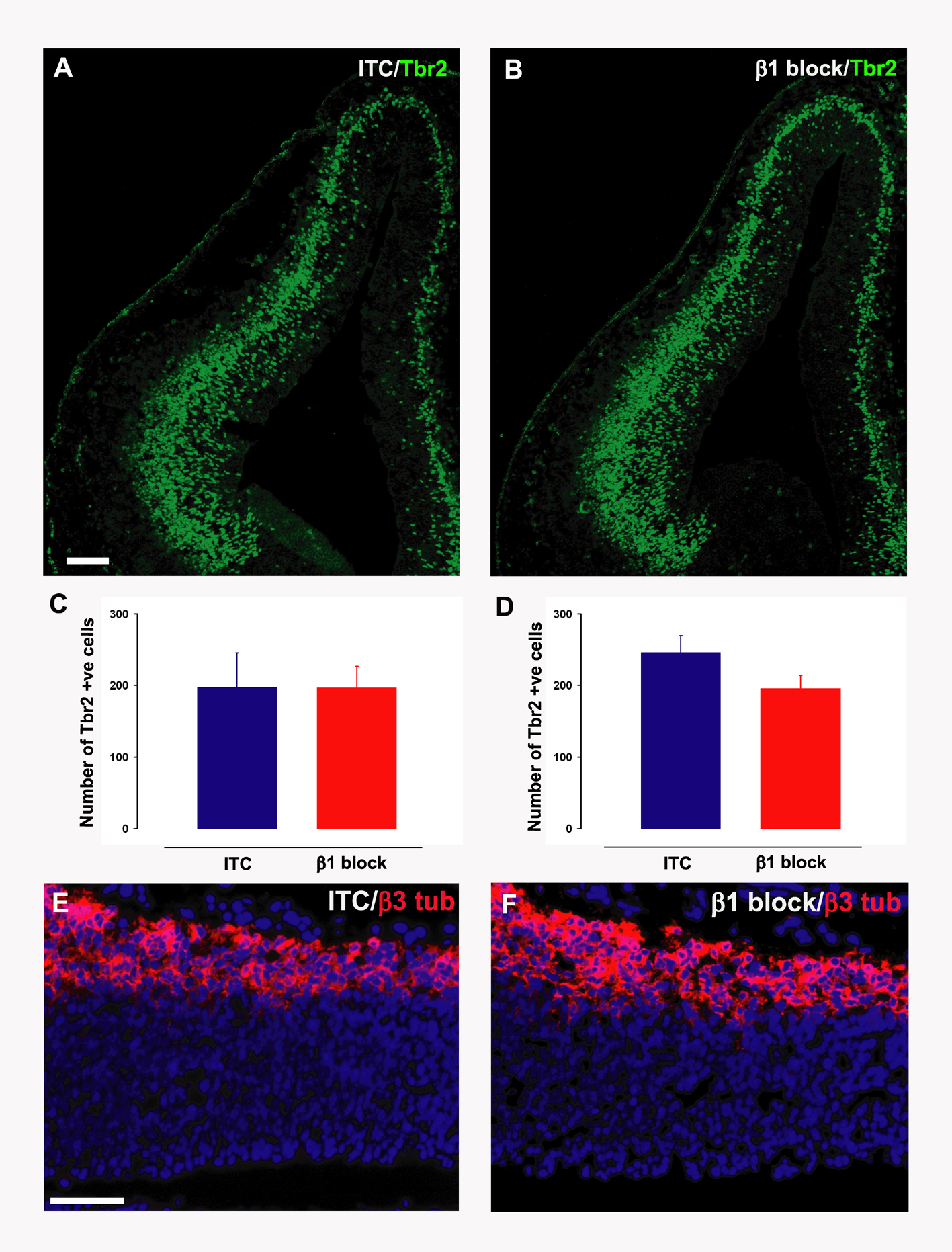

Supplement: Figure S4 — Cell differentiation is not affected after disruption of β1 integrin signalling at the ventricular surface. (A, B) Fluorescence micrographs of Tbr2 expression (green) after ITC (control, A) or β1 integrin blocking (B) antibody injection at E12.5. (C, D) Quantification of Tbr2+ cells in a dorsal (C) and ventral (D) 200 µm×100 µm (width×height) area of the VZ shows that the number of intermediate progenitors is not modified by the blockade of β1 integrin in the VZ. (E, F) Fluorescence micrographs of β3 tubulin expression (red, dapi-counterstained nuclei in blue) after antibody injection at E12.5. Note the lack of β3 tubulin expression within 100 µm of the ventricular surface; scale bar represents 50 µm. (7.92 MB TIF) [file pbio.1000176.s004.tif]

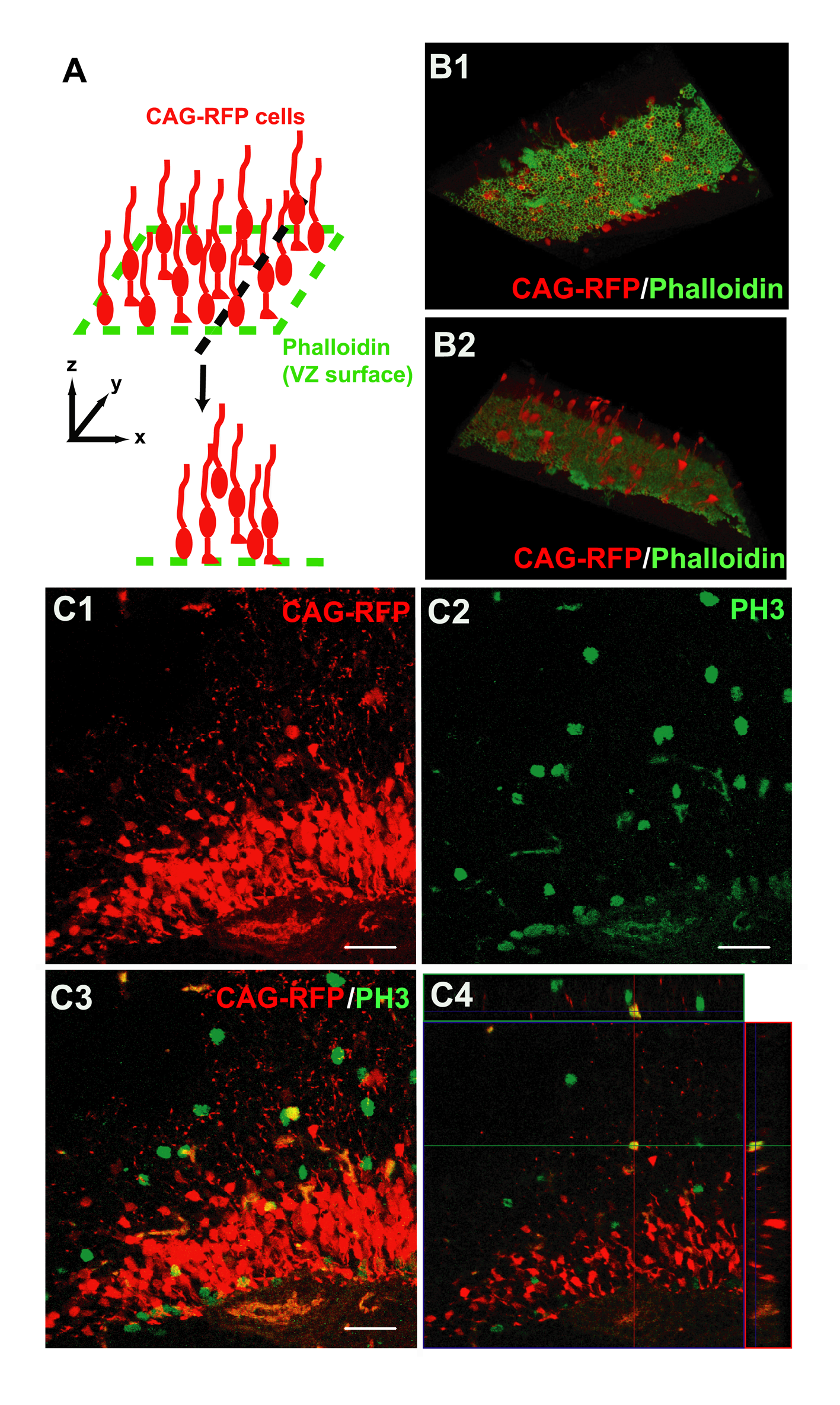

Supplement: Figure S5 — Intraventricular co-electroporation/injection of β1 integrin blocking antibody reveals detached cells. (A) Schematic representation of the delamination analysis including orthogonal sections to view the cortex from apical to basal surface followed by volumetric reconstruction and counting of the number of apical processes and cell bodies. (B) 3-D reconstruction pictures of CAG-RFP electroporated cells (in red) at the VZ surface (labelled with phalloidin in green) of E15.5 β1 integrin blocking antibody-injected brain. (C) Confocal fluorescence micrographs of E16 telencephalon stained with PH3 (C2, green) following β1 integrin blocking antibody and electroporation with CAG-RFP (C1, red) at E15.5. Note the co-expression of CAG-RFP and PH3 in a cell (yellow, C3, C4) away from the ventricular surface. Scale bar represents 50 µm. (10.08 MB TIF) [file pbio.1000176.s005.tif]

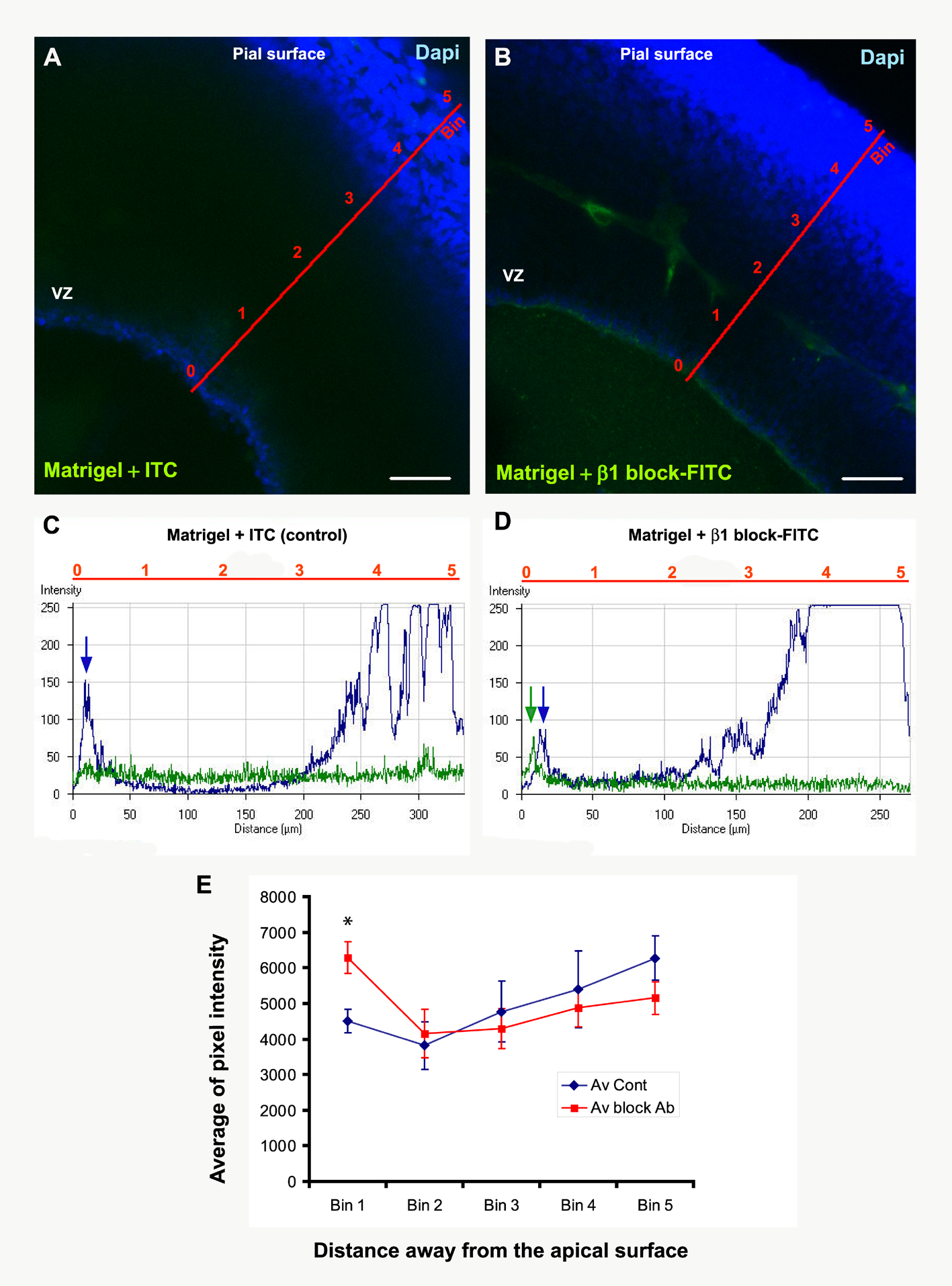

Supplement: Figure S6 — Analysis of the mobility/diffusion of the β1 integrin blocking antibody in growth factor-reduced matrigel during time lapse imaging experiments. (A, B) Fluorescent micrographs of 300-µm-thick neocortical E14.5 slices stained with dapi (blue) and containing a drop of ITC-(A) or FITC- conjugated β1 integrin blocking antibody (B) growth factor-reduced matrigel (GFRM) within their ventricular space. (C ,D) Intensity profile graphs illustrating the distribution of pixel intensity on both blue (dapi) and green (FITC antibody) channels from the VZ surface (bin 0) towards the pial surface (bin 5) with both ITC- (C) and β1 integrin blocking antibody FITC-conjugated (D) GFRM. Each bin represents 20% of the neocortical wall. Note that a green peak (green arrow) is only detected in the first bin of the β1 integrin blocking antibody FITC-conjugated GFRM (D), whereas a blue peak (blue arrows) corresponding to dapi at the apical surface of the neocortical slices is detected in both conditions. (E) Graph illustrating the average of green channel pixel intensity summed for each bin along the neocortical wall in β1 integrin blocking antibody FITC-conjugated GFRM (red line) compared to controls (blue line). Note that the pixel intensity in the first bin (corresponding at the apical surface) is significantly higher with the β1 integrin blocking antibody than in the control confirming that the antibody can diffuse into at least in the first 20% of the thickness of the neocortical wall. Conversely, the levels of antibody are not different from the control (noise/background) in bin 5 (that corresponds to the pial surface) indicating that the β1 integrin blocking antibody does not reach the pial surface. n = 6 cortices for ITC; n = 10 for β1 blocking antibody, ±SEM, *, p<0.05; unpaired two-tailed t-test. Scale bar represent 50 µm. (8.14 MB TIF) [file pbio.1000176.s006.tif]
